# IBD considerations in spondyloarthritis

**DOI:** 10.1177/1759720X20939410

**Published:** 2020-07-09

**Authors:** Caroline Di Jiang, Tim Raine

**Affiliations:** Department of Gastroenterology, Addenbrooke’s Hospital, Cambridge, UK; Department of Gastroenterology, Cambridge University Hospitals NHS Foundation Trust, Addenbrooke’s Hospital, Cambridge CB2 0QQ, UK

**Keywords:** crohn’s disease, inflammatory bowel disease, spondyloarthritis, ulcerative colitis

## Abstract

Spondyloarthritis (SpA) may be regarded a family of auto-inflammatory conditions with inflammation focused on the joints. These form part of a wider family of immune-mediated inflammatory diseases, which include inflammatory bowel diseases (IBD). These conditions share common elements of pathophysiology and it is perhaps unsurprising, therefore, that individuals with SpA frequently manifest gastrointestinal inflammation, to which the physician managing the patient with SpA must be alert. In this article, we review the shared epidemiology and pathophysiology of these conditions, before discussing approaches to diagnosis and management of inflammatory gastrointestinal pathology in patients seen in rheumatology clinics. In particular, we discuss the difference between non-specific gastrointestinal inflammation commonly described in this patient group and the more specific diagnosis of Crohn’s disease or ulcerative colitis. We describe the appropriate diagnostic workup for patients suspected of having IBD. In addition, we discuss how a diagnosis of IBD can inform treatment selection, highlighting important differences in treatment choice, drug dosing, monitoring and drug safety for this particular comorbid patient population.

## Introduction

Spondyloarthritis (SpA) refers to a family of chronic immune-mediated inflammatory joint diseases which share common genetic, pathophysiological and clinical features. One of their distinguishing characteristics are frequent extra-articular manifestations (EAMs).^1–3^ SpAs have been historically subcategorized into discrete disease entities, including ankylosing spondylitis (AS), psoriatic arthritis (PsA), and enteropathic arthritis. However, there is increasing recognition of these as overlapping diseases on a continuum, and the ASAS (Assessment of SpondyloArthritis Society) criteria now classifies SpAs based on clinical features, either as axial SpA (axSpA) or peripheral SpA (pSpA). ASAS also recognises the prevalence and significance of inflammatory bowel disease (IBD) in SpA and have incorporated coexisting IBD as a classification criterion.^2,3^

IBD is a group of chronic relapsing-remitting inflammatory conditions including Crohn’s disease (CD), ulcerative colitis (UC), and IBD-unclassified (IBD-U). The overlap between SpA and IBD is well established, and epidemiological studies have consistently shown a strong association between these diseases. This is particularly well described in the IBD literature, where radiological sacroiliitis is present in 20–50% of patients and progressive AS in 1–10%.^4,5^

## Incidence and prevalence

SpA is associated with macroscopic (endoscopic) gastrointestinal (GI) inflammation in 30–44%^5–8^ and microscopic (histologic) inflammation in 46–66% of cases.^6,7,9,10^ Higher joint activity scores [both the Bath Ankylosing Spondylitis Disease Activity Index (BASDAI) and Bath Ankylosing Spondylitis Metrology Index (BASMI)] are independently predictive of microscopic GI inflammation.^9^ There is a mismatch between patients’ clinical symptoms and their GI inflammatory activity, and even macroscopic lesions are frequently asymptomatic.^5^ Some studies have labelled this phenomenon as ‘silent IBD’, although this terminology is misleading, as there is insufficient evidence to suggest these asymptomatic lesions are definite precursors to overt IBD.

A minority of patients with subclinical GI inflammation do develop IBD over time, with a predisposition for CD. Cohort studies and meta-analyses quote a lifetime IBD risk of between 4% and 14% in patients with SpA.^1,10–13^ A prospective Belgian study of 123 patients with SpA followed over a mean period of 62 months found 7% of patients developed *de novo* IBD.^10^ This study also found articular remission to be protective against IBD, and chronic microscopic GI inflammation to be a risk factor, although even in the higher-risk subgroup only 13% of patients developed IBD during the follow-up period.^10^ A large population-based matched cohort study of 4101 AS patients matched with 28,591 controls found 4% of patients had a pre-existing diagnosis of IBD, increasing to 7.5% at the end of 20-year follow up.^13^

## Pathogenesis

There are clinical, genetic, immunologic and environmental links between SpA and IBD. The clear epidemiological association between these two diseases suggests a shared pathogenesis with different organ manifestations of a common inflammatory pathway. At a genetic level, there is shared heritability and familial clustering.^5^ Genome-wide association studies (GWAS) have identified shared loci associated with risk of development of both SpA and IBD, including association signals in or near to genes in the IL12/23 pathway.^4,14–17^ The associations between polymorphisms, gene expression and disease are complex and may differ for the same mutation between two diseases.

On a broad level, there are two theories to how joint and intestinal inflammation are mechanistically linked. These theories are not mutually exclusive and may overlap. The ‘causal’ hypothesis suggests articular inflammation is dependent on the extension of the immune response from the GI tract, and vice versa. Based on this hypothesis, luminal epithelial inflammation initiates an inflammatory cascade with subsequent systemic translocation of immune complexes and a ‘gut–joint migration’ inflammatory chain due to shared trafficking systems.^15,17,18^ The inverse may also be true, and synovial inflammation may have downstream GI effects. On the other hand, the ‘comorbid’ hypothesis suggests independent inflammatory processes in the two organ systems coexist due to common shared genetic risk factors and shared environmental modifiers. The inflammatory events may still perpetuate each other indirectly, for example, increased microbial product translocation due to increased gut permeability may augment distant articular inflammation.^17,18^ One important environmental modifier is the gut and skin microbiome, but their exact roles in the shared pathogenesis of SpA and IBD is yet to be defined. It is unclear whether dysbiosis is the cause or effect of pathogenic inflammation; nor are their effects on barrier function and immune modulation well understood.^1,18^

## Extra-articular manifestation or IBD?

Given the complex shared genetic, immunological, and environmental factors which drive gut and joint inflammation, SpA-EAM and IBD can be regarded as being on a continuum of GI inflammation ranging from asymptomatic microscopic inflammation to severe phenotypic manifestations. There are no data on whether subsets of GI inflammation mimic joint activity, while others are independent of joint activity. A pragmatic approach to management would be to regard most incidental microscopic GI change as not mandating intervention. The label of IBD should be reserved for clear evidence of characteristic GI inflammation associated with symptoms, particularly when these exhibit a longitudinal course independent of joint disease.

## Diagnosis

Consideration for potential concomitant IBD should be performed at time of rheumatological consultation. This evaluation should include a GI history with attention paid to recent changes in bowel habit, weight loss and rectal bleeding. Nocturnal passage of stool, in particular, can be helpful to differentiate IBD from functional GI disturbances, which are more associated with bloating and abdominal discomfort relieved by defecation. Risk factors for IBD include a family history of UC or CD. While smoking is a risk factor for developing CD, UC is more frequently seen in non-smokers and can present after recent smoking cessation. A drug history, including use of non-steroidal anti-inflammatory drugs (NSAIDs), is important (see the following). Focused abdominal examination evaluating for tenderness or a palpable abdominal mass should be undertaken and extended to include perianal examination where the patient reports symptoms of perianal discharge, pain or abscess which might suggest perianal fistulae.^19,20^

There is no universal diagnostic test for IBD. Instead, the diagnosis is made by correlating clinical, biochemical, radiological, endoscopic and histological features. International guidelines recommend that patients with clinically suspected IBD undergo screening with inflammatory markers, stool microscopy and culture, as well as faecal calprotectin (FC).^19,20^ Calprotectin is released by activated, degranulating neutrophils into the stool and hence is a marker of increased neutrophil translocation into inflamed intestinal mucosa. In a general population where the pre-test probability of IBD is low, the test serves as a useful non-invasive tool for differentiating inflammatory from non-inflammatory conditions, such as functional GI disorders.^21^ European Crohn’s and Colitis Organization guidelines advise against routine genetic testing, or serological tests such as p-ANCA or ASCA, due to limitations in test interpretation and sensitivity.

## Calprotectin

In contrast to its use in a general population, the utility of calprotectin as a screening tool for IBD in patients with SpA is less well established. In particular, the high prevalence of GI inflammation in this group results in decreased test performance. A systematic review evaluated the role of screening FC in patients with SpA.^22^ Elevated FC was observed in all seven observational studies included in the review and ranged from 21.2% to 70.7%. This was in turn associated with microscopic inflammation in 41.7–100% and macroscopic inflammation in 11–80% of cases. Two of the observational studies identified specific FC cutoffs and calculated their predictive value for IBD. Using FC cutoffs of 132 mg/kg and 266 mg/kg, the studies quoted a test sensitivity of 66.7% and 100%, respectively. Corresponding specificity was 76.9% and 78.7%.^5,23^ However, numbers analyzed were very low. The study quoting 100% sensitivity identified only three patients (incidence 1.5%) with IBD at the time of study conclusion.

This lack of reliable test sensitivity calls into question the utility of FC as a screening test in patients with SpA. For patients with a clinical history of symptoms suggestive of IBD, we prefer to proceed directly to endoscopic examination. Likewise, given the high rates of asymptomatic minor GI inflammation discussed previously which may be associated with elevated FC, and the fact that there is no evidence to support escalating or changing treatment based on GI inflammation alone in this subgroup, we do not believe that elevated FC in an otherwise asymptomatic patient should mandate an endoscopy. The only possible exception to this would be in the asymptomatic patient in whom an interleukin 17 (IL-17) inhibitor is being considered (see discussion in the following), where we propose elevated FC might serve as a screening test for potential need for further endoscopic evaluation.

## Endoscopy and VCE

Ileocolonoscopy remains the cornerstone of diagnostic evaluation of IBD, and it is important that a full lower GI endoscopy includes evaluation of the terminal ileum. Although intubation of the terminal ileum can be technically demanding and occasionally time consuming, the potential for isolated terminal ileitis in CD means an examination limited to colonic evaluation alone should be considered incomplete.^19,20,24^ Terminal ileal inflammation can scar and distort the ileocaecal valve, hence in the hands of an experienced endoscopist, unsuccessful terminal ileum intubation should raise concerns regarding potentially occult terminal ileitis. Mucosal biopsies should be taken from all macroscopically involved areas visualized. Histopathological assessment of mucosal biopsies should be correlated to endoscopic findings and is helpful in differentiating IBD from other forms of GI inflammation (including drug-induced, ischaemic and infectious aetiologies), subclassifying IBD type, and assessing disease activity.

Imaging with magnetic resonance imaging (MRI), computed tomography enterography, and/or transabdominal ultrasonography are useful complementary tools in staging Crohn’s, particularly for examination of the terminal ileum, more proximal small bowel, and detecting complications such as strictures and fistulae. Upper GI tract involvement can occur in 16% of patients with CD but routine gastroscopy is not recommended in adult patients in the absence of symptoms suggestive of proximal disease, due to the extremely low prevalence of isolated upper GI tract disease.^24,25^

Video capsule endoscopy (VCE) involves the patient swallowing (or receiving an endoscopically placed) wireless camera which transmits images to a receiver unit that the patient carries for several hours. Provided there are no significant structural abnormalities such as a stricture, the camera is passed into the stool and is not recovered. VCE is extremely sensitive at detecting small bowel mucosal abnormalities, but there is no opportunity to obtain tissue for histopathology and there is poor test specificity. Over 10% of healthy patients will have mucosal erosions seen on VCE which are clinically insignificant.^26^ If histologic evaluation is required, balloon-assisted enteroscopy can be used for regions inaccessible *via* standard endoscopy.^24^ In addition, with VCE there is a risk of provoking small bowel obstruction in a patient with a stricture.

There is nonetheless interest in the use of VCE as a non-invasive diagnostic test in SpA patients. The SpACE Capsule Study was a prospective trial comparing the diagnostic yield of VCE with standard ileocolonoscopy in detecting CD in SpA patients. VCE detected a much higher rate of small bowel inflammation (SBI) compared with ileocolonoscopy (42.2% *versus* 10.9%). SBI was graded using the Lewis Score, which is a validated VCE score for use in CD patients but not for patients with SpA. Small bowel enteroscopy and histologic evaluation was not performed, and a diagnosis of CD was made on the basis of VCE findings. Within this study, a positive VCE result led to change of management (beyond NSAID cessation alone) in 65% of cases. The authors of SpACE concluded that CD was more common than previously reported, and VCE had a superior detection rate compared with colonoscopy.^5^ However, the clinical relevance of the study findings should be questioned. In particular, the inference that CD can be diagnosed from SBI in SpA is not evidence based, especially in the absence of histology. There is also no evidence to suggest that asymptomatic SBI should alter clinical management. For these reasons, there is insufficient evidence to recommend routine VCE over standard ileocolonoscopy, and it is best reserved only for patients with a high level of clinical suspicion despite normal ileocolonoscopy and radiological imaging.

## Alternative imaging

Many patients with SpA, especially axSpA, will already have undergone lumbosacral MRI, the gold standard imaging modality for assessing axial or sacroiliac inflammation. There are some data on the prevalence of extraspinal incidental findings (IF) seen on lumbar spine MRIs performed in non-SpA cohorts. A large cohort study from 2013 retrospectively reviewed 3000 MRIs, but the only GI IFs found were colonic diverticulosis in 20.4% cases.^27^ A 2015 study retrospectively reviewed 1278 MRIs and only found one case of colonic diverticulosis and no additional GI IFs.^28^ There are no studies specifically examining IFs on lumbar spine MRIs performed for SpA patients, who would be expected to have much higher rates of small bowel abnormalities or concurrent IBD. There are also no data on the quality or quantity of small bowel visualized on spinal MRIs. Anecdotal evidence would suggest that in the absence of the oral contrast required for small bowel distension and visualization during dedicated small bowel MRI, the small bowel would not be adequately assessed, and further dedicated GI imaging is indeed necessary.

## Treatment

When managing SpA patients with concurrent IBD, there are specific treatment considerations; in particular, given the shared elements of pathogenesis highlighted previously, there is a degree of overlap in potential treatment options. However, not all treatments are effective for both conditions, and doses, monitoring, co-prescription, drug safety profiles and reimbursement may all vary according to the indication. Therefore, effective communication between rheumatologists and gastroenterologists is key for optimized management, and ideally an interdisciplinary approach or combined clinics should be utilized, as recommended by European League Against Rheumatism (EULAR).^29^

## NSAIDs and Cox-2 inhibitors

NSAIDs or cyclo-oxygenase (COX) inhibitors inhibit COX, isozymes that catalyse the formation of prostanoids from arachidonic acid. Prostanoids consist of prostaglandins, thromboxane and prostacyclin, and are pro-inflammatory mediators. NSAIDs thus exhibit analgesic and anti-inflammatory effects, which make them highly effective in treating rheumatological symptoms and disease.^30^

Through inhibition of prostaglandin production, NSAIDs also impair colonic barrier function by altering epithelial permeability, cell proliferation, and mucus production. They can produce *de novo* ulceration and mucosal injury throughout the GI tract.^30,31^ An association between COX inhibition and IBD relapse was first identified in a case report in 1981.^32^ Since then, numerous case reports have suggested a possible link between NSAID use and IBD exacerbation.

COX has two isozymes, COX-1 and COX-2. The COX-1 pathway is implicated in the maintenance of GI mucosal defence, whereas the COX-2 pathway is more specific to inflammation. Hence selective COX-2 inhibition should have a good GI safety profile and should not cause mucosal injury or IBD exacerbation. However, some studies have also suggested that COX-2 expression is upregulated in inflamed colonic mucosa to repair epithelial damage and regulate homeostasis.^30^ Hence COX-2 inhibition may also in theory interfere with colonic repair mechanisms.

Several epidemiological studies have examined the safety of COX-2 inhibition in IBD. A Cochrane systematic review found only two high-quality prospective trials assessing the effects of COX-2 inhibitors on IBD exacerbation.^33^ Trials on COX-2 inhibitors that have since been withdrawn from the market (due to side-effect profile) were excluded from review. The first of these studies found celecoxib treatment up to 14 days did not increase risk of clinical or histological relapse of UC.^34^ The second study found etoricoxib treatment up to 3 months did not increase risk of clinical relapse in IBD.^35^ Furthermore, the latter study found that all GI side effects were reversible after the COX-2 inhibitor was stopped. Although evidence was sparse, the Cochrane review concluded that there is no evidence COX-2 inhibition increased IBD exacerbation.

A more recent systematic review further examined association between IBD flares, COX-2 inhibitors, and non-selective NSAID use. This review included 18 prospective and retrospective trials, and again found no association between IBD flares and COX-2 inhibitors. Interestingly, on meta-analysis of non-selective NSAIDs, there was only a slight trend for IBD relapse, which was not statistically significant (relative risk 1.29, 95% confidence interval 0.92–1.80). Despite the substantial heterogeneity across the 18 studies, this systematic review concluded that both COX-2 and non-selective COX inhibitors appeared safe in IBD patients and should not be withheld.^36^

In summary, current evidence consistently demonstrates the safety of COX-2 inhibitors in IBD, especially when used in the short term, up to 3 months. Despite early case reports suggesting potential for harm, meta-analyses have not demonstrated risk of disease flare even with non-selective NSAIDs, although results are drawn from heterogenous studies. In the absence of higher-quality evidence, and referring to underlying pathophysiology, it would seem that where short courses of therapy are required, non-selective NSAIDs may be acceptable for IBD patients in good remission.^4^ Where more frequent or sustained use is required, or for higher-risk patients with more active IBD, COX-2 inhibitors should be considered.

## DMARDS

Current ASAS–EULAR guidelines recommend against routine use of conventional synthetic disease-modifying anti-rheumatic drugs (DMARDs) in axSpA, and recommend biological DMARDs including tumour necrosis factor (TNF) inhibitors and IL-17 inhibitors for persistent inflammatory activity.^37^ This differs from pSpA, where rapid initiation of conventional synthetic DMARDs are recommended, followed by biological DMARDs including TNF inhibitors, IL-17 inhibitors, IL-12/23 inhibitors, or Janus kinase (JAK) inhibitors if treatment target is not achieved.^38^ Only some of these DMARDs are effective in IBD, with variable efficacy even within the same biologic subclass (e.g. TNF inhibitors).

Methotrexate is effective in maintaining clinical remission in CD but is ineffective at inducing remission^39^ and has no role in the treatment of UC.^40^ Sulfasalazine is sometimes used for psoriatic arthritis, although generally considered to be less effective compared with other DMARDs. It is metabolized in the colon to mesalazine, which is effective in inducing and maintaining remission in mild-to-moderate UC, especially in moderate-to-high doses.^41,42^ There is no evidence that leflunomide is effective in IBD.

Although thiopurines have no role in the management of SpA, they are frequently used in a broad range of indications in IBD, and their efficacy documented from numerous prospective trials. A 2016 systematic review demonstrated robust evidence in thiopurines inducing remission in IBD when used as combination therapy with an anti-TNF, as well as in maintaining remission in CD and preventing post-op CD recurrence when used as monotherapy.^43^ The levels of active thiopurine metabolites required to prevent anti-TNF antibodies have not been well defined, but are likely lower than levels required to achieve disease remission when used as monotherapy.^44,45^

Infliximab is effective in inducing and maintaining remission in UC evidenced in the ACT 1 and ACT 2 trials,^46^ as well as in CD evidenced in the ACCENT 1 trial.^47^ Conventional dosing in IBD is 5 mg/kg at weeks 0, 2, 6 and then 8 weekly thereafter. The SONIC trial demonstrated infliximab in combination with 2.5 mg/kg azathioprine was more successful in achieving clinical and endoscopic remission compared with infliximab monotherapy at week 26.^48^ Subsequently, studies have shown immunomodulators (such as thiopurines and methotrexate) improve the pharmacokinetics of infliximab and are associated with reduced infliximab clearance.^49^ The PANTS study found low anti-TNF drug levels at week 14 predicted non-remission at week 54, commonly due to the development of immunogenicity and presence of anti-TNF antibodies. The addition of an immunomodulator mitigates the risk of immunogenicity hence risk of treatment failure.^50^ A systematic review in 2018 found infliximab had considerably greater immunogenicity compared with adalimumab,^51^ highlighting the role of co-prescription of infliximab with an immunomodulator. There is increasing evidence in the IBD literature on the use of therapeutic drug monitoring (TDM) through infliximab trough measurements, and dose optimization to reach a target trough range. This can be achieved by increasing infliximab dose to 10 mg/kg and/or shortening infusion intervals to a minimum of 4 weeks. The TAXIT and TAILORIX trials compared dose optimization based on TDM with clinical features alone and found no difference in rates of clinical remission at 1 year.^52,53^ However, the TAXIT trial demonstrated fewer IBD flares in the TDM group. There is also increasing evidence that TDM leads to most cost-effective use of infliximab.^54^ Subcutaneous infliximab has recently been approved for use in rheumatoid arthritis and is the subject of an encouraging switch study in IBD. Results from 1 year suggest similar efficacy and safety compared with intravenous infliximab, with potentially favourable pharmacokinetics.^55^ Phase III trials are ongoing, and subcutaneous infliximab may prove to be an attractive future treatment option for IBD.

Adalimumab is effective in inducing and maintaining long-term remission in UC, evidenced in the ULTRA1, ULTRA2 and ULTRA3 trials,^56–58^ as well as in CD, evidenced in the CLASSIC 1, CHARM and EXTEND trials.^59–61^ The typical induction regime used in IBD is 160 mg at week 0 followed by 80 mg at week 2. Escalation of maintenance treatment from 40 mg fortnightly dosing to weekly dosing can be helpful in recapturing response. In the ULTRA 2 trial, weekly adalimumab was given to 38% of week 8 non-responders, resulting in clinical improvement without new safety signals.^57,62^ Unlike infliximab, meta-analyses have shown no benefit in adding an immunomodulator to adalimumab in inducing or maintaining remission.^63^ The exception to this, however, is when a patient is switched to adalimumab after developing infliximab antibodies, given the higher risk of *de novo* adalimumab antibodies in this group.^64^ A recent randomized controlled trial has demonstrated IBD patients with immune-mediated loss of response to anti-TNF have improved clinical outcomes when azathioprine is added to their second anti-TNF. The majority of patients in this study were switched from infliximab to adalimumab.^65^

Certolizumab pegol contains only the antigen-binding fragment (Fab) of the monoclonal antibody, hence has unique properties such as lack of trans-placental transfer.^66^ It is effective in the induction and maintenance of CD, evidenced in the PRECiSE 1 and PRECiSE 2 trials, up to 26 weeks follow up.^67,68^ In PRECiSE 1, although certolizumab was superior to placebo in achieving clinical response, remission rates did not differ significantly.^67^ Currently, certolizumab is only licensed for use for CD in Switzerland and the USA. There are very limited data on its use in UC, and it is not approved for this indication.^69^ Golimumab is licensed for use in UC, with the PURSUIT-SC and PURSUIT-M trials demonstrating its success in inducing and maintaining UC remission.^70,71^ It is not currently approved for use in CD and there are few studies examining its efficacy for this indication. A French retrospective study of 154 patients observed clinical response in 56% of CD patients after a mean treatment duration of 4 months.^72^ A Swiss case series used golimumab as a fourth-line anti-TNF in eight refractory CD patients. Five patients responded to induction therapy, and three of these patients had a sustained clinical response.^73^ In contrast, etanercept had no efficacy in the treatment of IBD.^74^ In fact, a Danish cohort study has demonstrated it is more likely than other anti-TNF agents to provoke *de novo* IBD when used for other autoimmune conditions.^75^

Ustekinumab is an anti-IL12/23 p40 antibody which is effective in pSpA, and has no efficacy in axSpA.^76^ It has been demonstrated as effective in the induction and maintenance of CD, evidenced by the UNITI and IM-UNITI trials,^77^ and in the induction and maintenance of UC, evidenced by the UNIFI trials.^78^ Induction and maintenance doses used in IBD are much higher compared with pSpA. Induction is a single intravenous infusion and weight based, with dose ranging between 260 mg and 520 mg. Maintenance therapy is 90 mg subcutaneous injections every 12 weeks (first dose 8 weeks after induction), with dosing frequency reduced to 8 weeks for higher-risk patients including partial responders, those with loss of response, or where there is history of anti-TNF treatment failure.

Tofacitinib is a potent JAK inhibitor which is highly effective at inducing and maintaining remission in UC, evidenced by the OCTAVE-1, OCTAVE-2 and OCTAVE-SUSTAIN trials.^79,80^ It has not been demonstrated effective in CD.

## Secukinumab

Secukinumab is a recombinant, fully human immunoglobulin G1k (IgG1k) monoclonal antibody that inhibits IL-17A, a pro-inflammatory cytokine produced by T-helper (Th) 17 cells. IL-17A has a crucial role in the pathogenesis of multiple immune-mediated inflammatory conditions; including psoriasis, SpA and IBD.^81,82^ By selectively targeting IL-17A, secukinumab has immunomodulatory effects, and has been demonstrated as highly efficacious in the treatment of both axSpA and pSpA.^83^ However, contrary to the initial hypothesis that IL-17 blockade would also improve IBD activity, a CD trial unexpectedly showed that secukinumab paradoxically worsened CD activity, and the trial was terminated prematurely.^84^ Similar results were replicated with brodalumab, an anti-IL-17 receptor antibody.^85^ Since then, there have been no further trials studying use of IL-17 inhibitors in IBD, but there has been increasing evidence suggesting IL-17 inhibition in SpA is associated with *de novo* IBD and exacerbation of pre-existing disease.

It is speculated that in contrast to its pro-inflammatory role in SpA, IL-17 may have a protective effect on intestinal mucosa and a role in GI homeostasis rather than driving pathogenic inflammation. IL-17A blockade may cause an imbalance and dysregulation of mucosal cytokines and may lead to the Th1 pathway being favoured. This induces GI tract mucosal inflammation and promotes IBD pathogenesis.^86,87^ A second hypothesis is that IL-17 inhibition changes the colonic fungal microbiome composition *via* unchecked *Candida albicans* proliferation, which predisposes susceptible individuals to developing *de novo* IBD.^88^

In 2019, Schreiber *et al*. conducted a large retrospective analysis using pooled data from 21 clinical trials to identify IBD incidence in patients receiving secukinumab, across indications of SpA and psoriasis. A total of 7355 patients were included, with a cumulative exposure to secukinumab of 16,227 patient-years. A total of 41 cases of IBD flares were identified (0.56%): 73% of cases were *de novo* IBD and the remainder were disease relapses. Analysis found low exposure-adjusted incidence rates (EAIRs) ranging between <0.1 and 0.4 per 100 patient-years for CD, UC and IBD-U. Compared with placebo-controlled treatment groups, the relative risk for developing IBD or having a relapse was not statistically significant.^89^

A large secukinumab safety analysis published in the same year combined data from this previous analysis with post-marketing surveillance data up to 5 years. Pooled data from approximately 112,000 patient-years identified low EAIRs ranging from 0.01 to 0.2 per 100 patient-years for CD, UC and IBD-U. The observed EAIRs did not increase over time with cumulative exposure.^83,89^ It is important to note that active IBD was an exclusion criterion for all secukinumab trials. Hence, the safety of secukinumab should not be extrapolated to patients with suspected or confirmed IBD activity.

Similar findings have been replicated in dermatology literature. A 2017 systematic review found similar rates of IBD flares when anti-IL-17 agents were used for psoriasis. All three anti-IL-17 agents studied (secukinumab, brodalumab and ixekizumab) produced similar rates of IBD, suggesting a class effect. Pooled data analysis from secukinumab studies (*n* = 3430) found EAIRs of 0.11 and 0.15 per 100 patient-years for CD and UC, respectively.^90^

In contrast to these retrospective analyses, a 2019 survey identified higher rates of *de novo* IBD in patients receiving secukinumab. The survey was conducted amongst dermatologists and rheumatologists in Italy. Of 434 patients treated with secukinumab over a 2-year period, four patients (approximately 1%) developed new-onset IBD. These rates may be more reflective of true disease incidences in the real world.^91^

Although the incidence of IBD flares or *de novo* IBD are low from these studies, care is still required when using secukinumab in patients with suspected IBD. There has been a case report describing rapid-onset fulminant colitis after a single dose of secukinumab infusion. The patient had no pre-existing GI symptoms or IBD diagnosis, and her only risk factor was a strong family history of IBD in first-degree family members.^86^ Hence, although the absolute risk for IBD flare might be low, the phenotype can be extremely aggressive.

In summary, there is an absolute contraindication for IL-17 blockade use in IBD patients with active disease. Given the low but serious risk of fulminant disease flare, it is also relatively contraindicated in patients with IBD in remission, uninvestigated GI symptoms or a strong family history. It seems prudent to assess all patients in whom IL-17 blockade is being considered. Those with uninvestigated GI symptoms should be assessed clinically and undergo lower GI endoscopy where appropriate. The presence of macroscopic, and probably microscopic, inflammation should prompt extreme caution for proceeding with IL-17 inhibition. Likewise, any patient with a first-degree relative having a diagnosis of IBD should be assessed, with either an ileocolonoscopy, or at least screening with FC and ileocolonoscopy if FC is elevated. All patients starting IL-17 blockade should be counselled to seek prompt medical attention if new GI symptoms develop, which should be, in turn, be thoroughly investigated.

## Vedolizumab

Vedolizumab is a gut-specific monoclonal antibody which binds to α4β7 integrin and selectively inhibits leucocyte adhesion to the vascular addressin MAdCAM-1, which is restricted to the intestinal endothelium. Vedolizumab reduces GI inflammation by preventing lymphocyte migration and translocation into the intestinal tract, and provides a highly targeted mechanism of action for IBD with minimal systemic adverse events.^92^ Unlike natalizumab, another α4-integrin monoclonal antibody which targets both α4β1 and α4β7 subunits, vedolizumab has no effect on the central nervous system, hence has no association with complications such as progressive multifocal leucoencephalopathy.^92^

Phase III GEMINI trials have demonstrated efficacy of vedolizumab in the induction and maintenance of both UC and CD.^93,94^ Patients with anti-TNF-responsive SpA but poorly controlled IBD may benefit from the addition of vedolizumab for their luminal disease. Rheumatology literature has suggested significantly increased adverse events when combining biologics; however, the limited systemic expression of α4β7 means vedolizumab should, in theory, have a favourable safety profile.^95^ A 2007 randomized controlled trial comparing infliximab plus natalizumab with infliximab monotherapy for refractory CD showed no excess safety signals, nor statistically significant improvement in outcomes.^96^ There are also two case reports and a case series of seven patients who received combination anti-TNF and vedolizumab, none of whom developed serious adverse events.^95,97^ Although data are currently very limited, this treatment strategy might be considered for select refractory patients who require an anti-TNF for SpA and vedolizumab for coexisting IBD.

*Post hoc* analyses from GEMINI trials found a significant reduction of arthralgia/arthritis (hazard ratio 0.63) in CD patients treated with vedolizumab compared with placebo; and comparable incidence of arthralgia/arthritis in UC patients. This is likely due to reduced extra-intestinal manifestations after achieving improved IBD control.^98^ However, vedolizumab has also been associated with rheumatological adverse reactions, ranging from self-limiting arthralgia to *de novo* SpA. A retrospective review of 71 patients receiving vedolizumab over 6 years identified eight patients who developed new or worsening arthralgia, of which vedolizumab was discontinued in five patients with resolution of arthralgia.^99^ There have also been two case series in 2016 (*n* = 5) and 2019 (*n* = 11) documenting severe SpA and/or enthesopathy after starting vedolizumab, with the majority being *de novo* cases.^100,101^ Median time between first dose of vedolizumab to SpA flare was 12 weeks, and 70% of cases had well controlled IBD at time of flare.

## Conclusion

The physician caring for the patient with SpA needs to be aware of the potential for inflammatory bowel pathology within this group. This has implications for clinical assessment, as well as for treatment selection. The mainstay of IBD diagnosis is clinical history, supported by appropriate ileocolonoscopic evaluation, and small bowel imaging where required. FC is a useful screening test for IBD in the general population but test performance may limit utility in patients with SpA. Suitable treatments for SpA include a subgroup that are effective for IBD, but dose selection and dose titration differ, with typically higher doses used in IBD and increased attention paid to dose escalation. NSAIDs and anti-IL-17 drugs, while useful in SpA, must be used with considerable caution in patients with IBD. GI side effects in patients on IL-17 inhibitors in particular, need to be thoroughly assessed. Given these complexities, effective communication between rheumatologists and gastroenterologists is imperative for optimal patient care.
